# Pyrroloquinoline-quinone to reduce fat accumulation and ameliorate obesity progression

**DOI:** 10.3389/fmolb.2023.1200025

**Published:** 2023-05-05

**Authors:** Nur Syafiqah Mohamad Ishak, Kazuto Ikemoto

**Affiliations:** Niigata Research Laboratory, Mitsubishi Gas Chemical Company, Inc., Niigata, Japan

**Keywords:** PQQ, pyrroloquinoline quinone, fat, obesity, lipogenesis, mitochondria, inflammation, metabolic syndrome

## Abstract

Obesity is a major health concern worldwide, and its prevalence continues to increase in several countries. Pyrroloquinoline quinone (PQQ) is naturally found in some foods and is available as a dietary supplement in its disodium crystal form. The potential health benefits of PQQ have been studied, considering its antioxidant and anti-inflammatory properties. Furthermore, PQQ has been demonstrated to significantly influence the functions of mitochondria, the organelles responsible for energy production within cells, and their dysfunction is associated with various health conditions, including obesity complications. Here, we explore PQQ properties that can be exploited in obesity treatment and highlight the underlying molecular mechanisms. We review animal and cell culture studies demonstrating that PQQ is beneficial for reducing the accumulation of visceral and hepatic fat. In addition to inhibiting lipogenesis, PQQ can increase mitochondria number and function, leading to improved lipid metabolism. Besides diet-induced obesity, PQQ ameliorates programing obesity of the offspring through maternal supplementation and alters gut microbiota, which reduces obesity risk. In obesity progression, PQQ mitigates mitochondrial dysfunction and obesity-associated inflammation, resulting in the amelioration of the progression of obesity co-morbidities, including non-alcoholic fatty liver disease, chronic kidney disease, and Type 2 diabetes. Overall, PQQ has great potential as an anti-obesity and preventive agent for obesity-related complications. Although human studies are still lacking, further investigations to address obesity and associated disorders are still warranted.

## 1 Introduction

Pyrroloquinoline-quinone (PQQ) was discovered as a bacterial coenzyme for dehydrogenase (Hauge, 1964), and its structure was later determined by derivatized crystallography (Salisbury et al., 1979). PQQ is also present in small amounts of everyday food, including fruits, vegetables, fermented foods, and breast milk (Kumazawa et al., 1995; Mitchell et al., 1999). PQQ is required for normal growth and maintenance (Killgore et al., 1989). Moreover, PQQ functions as a vitamin (Kasahara and Kato, 2003) and is vital for good health (Jonscher et al., 2021).

Redox reactions are essential to PQQ given their quinone structure. PQQ can be reduced by various substances and converted back to its oxidized state by oxygen. This reaction describes how PQQ functions as the active site of glucose dehydrogenase and can be used as a glucose sensor to manage diabetes (Teymourian et al., 2020). Furthermore, PQQ functions as an antioxidant. Although PQQ in food is in its oxidized form, it is easily reduced by reacting with biological materials. Reduced PQQ scavenges free radicals and reduces oxidative stress, causing cellular damage and contributing to developing chronic diseases (Ouchi et al., 2013). The antioxidant capacity of PQQ contributes to its longevity and anti-inflammatory effects (Sasakura et al., 2017; Yin et al., 2019). Moreover, owing to its ability to increase nerve growth factor production (Yamaguchi et al., 1996) and protect against oxidative damage and encephalitis (Scanlon et al., 1997), PQQ improves cognitive function (Itoh et al., 2016; Tamakoshi et al., 2023).

PQQ can stimulate new mitochondrial growth in cells by increasing peroxisome proliferator-activated receptor-gamma coactivator 1 alpha (PGC-1α) and activating the transcriptional networks (Chowanadisai et al., 2010). Such signaling, often including the AMP-activated protein kinase (AMPK) pathway, is linked to cellular increases in the NAD+/NADH ratio and increased sirtuins expression. In this regard, PQQ improves energy regulation, where genes essential for fatty acid metabolism and mitochondrial function are particularly enhanced (Jonscher et al., 2021). Thus, PQQ could be an alternative food ingredient to prevent obesity. However, human studies on the treatment of obesity with PQQ are lacking.

PQQ was launched as a food ingredient in the United States in 2008. Mitsubishi Gas Chemical Co., Ltd. produced PQQ as pyrroloquinoline quinone disodium (product named BioPQQ^®^, C_14_H_10_N_2_Na_2_O_11_, formula weight: 428.22 amu) by fermentation. PQQ has been certified as a functional food and a new dietary ingredient. In 2014, the Japanese Ministry of Health, Labour, and Welfare approved it as a health food ingredient. In addition, the European Commission approved the product in 2018 as a novel food ingredient named MGCPQQ^®^, after passing the European Food Safety assessment (Turck et al., 2017). Long-term human experiments with PQQ have not revealed complications, making it food-grade. The product, disodium 9-carboxy-4,5-dioxo-4,5-dihydro-1H-pyrrolo[2,3-f] quinoline-2,7-dicarboxylate, was a crystalline red trihydrate (Ikemoto et al., 2012) distinct from the free form, 4,5-dioxo-4,5-dihydro-1H-pyrrolo[2,3-f] quinoline-2,7,9-tricarboxylic acid. The free form does not exist in nature, and its physical properties are distinct from those of the food ingredient disodium salt (Ikemoto et al., 2017). However, there is no distinction in the use of free-form PQQ and PQQ disodium in most academic literature and in the present review.

Obesity and its related co-morbidities are increasing globally. Hence, finding a therapeutic agent to prevent and treat obesity is crucial. In this review, we specifically focus on the properties of PQQ that can be used in obesity management.

As PQQ supplementation has been studied for myriad potential health benefits (Akagawa et al., 2016), here we described the findings of recent studies on how it ameliorates obesity-related diseases.

## 2 Factors contributing to obesity development

Obesity is a medical condition and is often the result of a complex interplay among genetics, the environment, developmental programming, and gut microbiome factors. Although numerous factors contribute to obesity, it is eventually caused by an energy imbalance. When more calories are consumed than burned, the excess energy is stored in the body as fat. Excess accumulated body fat causes mitochondrial dysfunction, inflammation, hyperlipidemia, and insulin resistance, leading to various metabolic health problems (Jung et al., 2014). Such conditions contribute to co-morbidities such as Type 2 diabetes, high cholesterol, and cardiovascular disease (Guh et al., 2009; Tsatsoulis and Paschou, 2020). Treating obesity often involves a combination of lifestyle changes, particularly improving diet and increasing physical activity, as well as medical interventions, such as drugs and surgery (Wiechert and Holzapfel, 2021). Here, we propose PQQ as a preventive measure against obesity and its associated health conditions. PQQ reportedly reduces fat accumulation (Mohamad Ishak et al., 2021), and alters lipid metabolism (Qiu et al., 2021; Zhang et al., 2022) and adipokine production (Devasani et al., 2020). Hence, herein, we discuss the effects of PQQ on obesity and its mode of action, primarily based on recent research based on multiple animal and cell culture studies.

## 3 PQQ reduces body fat accumulation to prevent obesity

**FIGURE 1:**
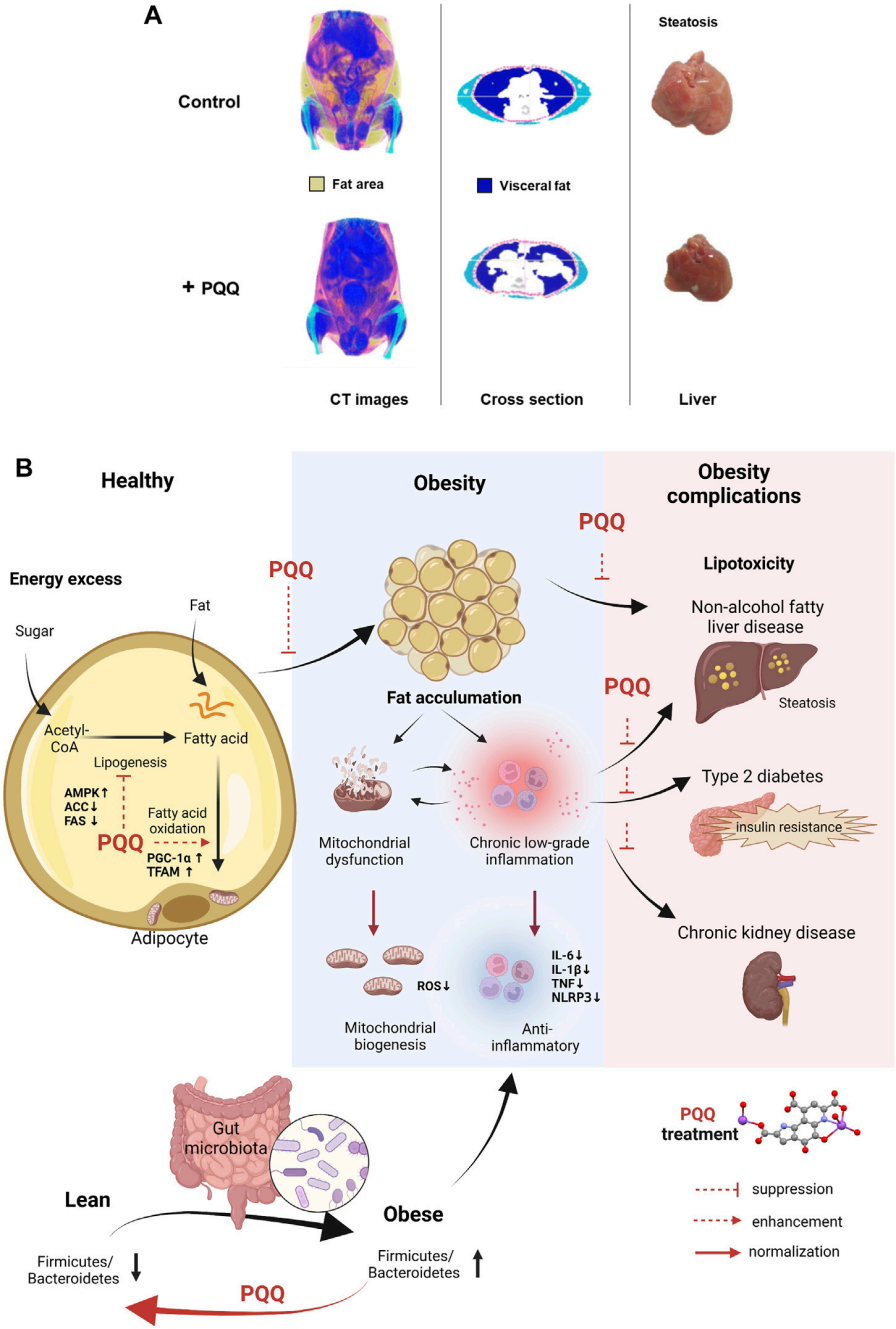
**PQQ in reducing fat accumulation and ameliorating obesity progression.** **(A)** PQQ reduced visceral and hepatic fats in HFD-induced obese mice. Control mice have accumulated fat, especially visceral fat. The steatosis condition caused by fats building up in the liver was observed in control mice. The images were obtained and edited from our previous study (Mohamad Ishak et al., 2021). **(B)** PQQ action in reducing fat accumulation and improving obesity conditions. Fat accumulation occurs when excess energy, such as sugar (glucose), is converted into fatty acids via lipogenesis and stored in fat cells (adipose tissue) as triglycerides, leading to obesity. When necessary, fatty acids are metabolized through beta-oxidation (or fatty acid oxidation) in the mitochondria, and energy is produced in the form of adenosine triphosphate. PQQ attenuates fat accumulation by suppressing fatty acid synthesis (lipogenesis) and enhances beta-oxidation by boosting mitochondrial biogenesis. Mitochondrial dysfunction and inflammation occur during obesity progression. Mitochondrial dysfunction causes ROS accumulation and activates inflammatory pathways, releasing pro-inflammatory cytokines and inflammasomes. Inflammation further impairs mitochondrial function, creating a cycle of dysfunction and inflammation. When inflammation becomes chronic, it can lead to tissue damage and contribute to the development of insulin resistance. These events cause various health conditions, including NAFLD, type 2 diabetes, and CKD. With PQQ treatment, the enhancement of mitochondrial biogenesis mitigates mitochondrial dysfunction complications and its anti-inflammatory properties aid in resolving inflammation. Additionally, PQQ improved insulin resistance, and alleviates lipotoxicity in the fetal kidney and liver by decreasing ROS levels. The anti-obesity effects of PQQ have been shown to improve obesity complications. The microbiota Firmicutes/Bacteroidetes (F/B) ratio refers to the ratio of two major phyla of bacteria in the gut microbiome. Studies have shown that an increased F/B ratio is associated with obesity and other metabolic disorders. PQQ reversed F/B ratio in HFD animals and reduced inflammatory markers. The illustration was created with BioRender.com.

Obesity can be evaluated based on body fat content because elevated visceral and hepatic fat levels increase the risk of developing chronic diseases (Thomas et al., 2012). We summarized animal and cell studies on PQQ roles in reducing body fat accumulation in Table 1. These results indicated that PQQ could attenuate body fat, especially visceral and hepatic fat accumulation to prevent dietary obesity (Figure 1A). As obesity progresses with increased fat cell number and size, increasing adipose tissue mass (Sakai et al., 2007), PQQ reduced lipid content and droplet size in mouse adipocytes (Mohamad Ishak et al., 2021). Moreover, in condition to exacerbate obesity and diabetes with benzyl butyl phthalate (BBP) exposure (Zhang et al., 2020), a common endocrine-disrupting chemical (EDC), PQQ normalized the increased liver weight in HFD- and BBP-treated male mice, suggesting that PQQ prevented hepatic fat accumulation. However, female mice showed inconsistent data owing to hormonal changes and reduced EDC susceptibility (Nickelson et al., 2012; Zhang et al., 2022).

**TABLE 1:** Summary of animal and cell culture studies and their key findings on PQQ roles associated to obesity.

Animal (Strain)	Culture conditions	PQQ treatment	Key findings	References
Male mice (C57BL/6J)	HFD (60% fat to calorie ratio)	20 mg/kg/day (6 weeks)	• PQQ significantly attenuated total body and visceral fat volume	Mohamad Ishak et al. (2021)
Female and male mice (C57BL/6J)	HFD (60% fat to calorie ratio) with and without BBP (3 mg/kg/day	20 mg/kg/day (16 weeks)	• PQQ significantly attenuated body weight gain, liver weight and diabetes condition in HFD- and BBP-treated male	Zhang et al. (2022)
			• PQQ restored metabolites level essential for mitochondrial beta-oxidation	
Male rats (Sprague Dawley)	HFD (60% fat to calorie ratio, 10% fructose content)	10 and 20 mg/kg/day (5 weeks after 10 weeks diet-induced obesity)	• PQQ significantly reduced intra-abdominal fat and liver weight *per se* and with ATS.	Devasani and Majumdar (2019), Devasani et al. (2020)
			• PQQ considerably improved serum lipid profile and glucose tolerance	
			• PQQ upregulated PCG-1α, SIRT1 and TFAM, augmenting mitochondrial biogenesis	
			• PQQ with ATS reduced inflammasome (NLRP3, caspase 1) and inflammatory markers (IL-1β, IL-18, IL-6)	
Hens (Hy-line)	HELP diet (metabolic energy = 12.75 MJ/kg, crude protein = 13%)	0.08 and 0.16 mg/kg (4 weeks)	• PQQ effectively reduced liver fat content, suppressing steatosis progression	Qiu et al. (2021)
			• PQQ improved serum lipid metabolism and anti-oxidative capacity	
Female mice and offspring (C567BL/6J)	Study 1 (maternal): HFD (45% fat to calorie ratio) Study 2 (offspring): WD (42% fat to calorie ratio, 34% fructose content)	3.8 µM PQQin drinking water (provided after mating or 12.2 µM after weaning)	• PQQ reduced body and hepatic fat of the offspring	Jonscher et al. (2017), Mandala et al. (2022)
			• Pre- and postnatal PQQ supplementation protects offspring from NAFLD progression	
			• PQQ showed long term protective effects on hepatic lipotoxicity and inflammation in obese mice	

Cell type (Line)	Culture conditions	PQQ treatment	Key findings	References
Mouse adipocytes (3T3-L1)	Cell differentiation with 10% fetal bovine serum, 0.5 mM 3-isobutyl-1-methylxanthine, 1 μM dexamethasone, 2 μM rosiglitazone, and 5 μg/mL insulin	0, 50, 100 or 200 nM of PQQ (48 h)	• PQQ reduced lipid content and droplet size	Mohamad Ishak et al. (2021)
			• PQQ suppressed lipogenesis by activated AMPK pathway which essential for regulating fatty acid synthesis	
			• PQQ upregulated PGC-1α and TFAM that promotes mitochondrial biogenesis	
Primary Chicken Hepatocytes	Steatosis-induced with 1 mM free fatty acid for 24 h and oxidative stress-induced with 4 mM H_2_O_2_for 4 h	0, 50, 100, 200 or 400 nM of PQQ (24 h)	• PQQ improved lipid metabolism anti-oxidative capacity, hepatic mitochondrial functions, and apoptosis signals	Qiu et al. (2021)

Adipose tissue stores excess energy inside fat cells as triglycerides (TG). When necessary, stored TG are broken down and released as free fatty acids outside the cells to provide energy. This process occurs in the mitochondria and involves a series of chemical reactions that break down fatty acids into acetyl-CoA (beta-oxidation). As PQQ is known to enhance mitochondrial function, it attenuated diet-induced fat accumulation primarily by improving beta-oxidation through mitochondrial biogenesis and suppressing lipogenesis (Figure 1B). PGC-1α is a critical factor that stimulates mitochondrial oxidative metabolism and promotes mitochondrial biogenesis (Wu et al., 1999; Puigserver and Spiegelman, 2003). Specific conditions such as caloric restriction and exercise can increase PGC-1α expression (Wu et al., 1999; Russell et al., 2003). PQQ enhances PGC-1α expression, eventually improving mitochondrial biogenesis (Chowanadisai et al., 2010; Saihara et al., 2017), suggesting that PQQ could also stimulate PGC-1α expression similar to exercise or caloric restriction. Recent diet-induced obesity studies also showed that PQQ supplementation enhanced mitochondria biogenesis by regulating PGC-1α, NRF1, and TFAM through the AMPK (adenosine monophosphate-activated protein kinase) signaling pathway (Devasani et al., 2020; Mohamad Ishak et al., 2021; Qiu et al., 2021). Moreover, studies revealed PQQ promoted lipid metabolism as PQQ treatment considerably reduced TG, total cholesterol, and low-density lipoprotein cholesterol levels in the serum lipid profile of diet-induced obese animals (Devasani and Majumdar, 2019) and hens (Qiu et al., 2021)and adipocytes (Mohamad Ishak et al., 2021). Diet-induced obesity in mice lowered acetyl-L-carnitine, propionylcarnitine, and stachydrine levels, metabolites essential for mitochondrial energy production. This condition was restored by PQQ treatment, suggesting beneficial metabolic improvement (Zhang et al., 2022). These results indicate that PQQ improves beta-oxidation and enhances mitochondrial biogenesis, reducing fat accumulation.

PQQ suppresses lipogenesis, through which the body synthesizes new fat molecules from non-fat sources. This process occurs primarily in the liver and adipose tissue and uses acetyl-CoA for fatty acid synthesis, forming TG (Semenkovich and Goldberg, 2020). PQQ treatment activated AMPK, which increased the phosphorylation of acetyl-CoA carboxylase 1 and sterol regulatory element-binding protein 1c in adipocytes (Mohamad Ishak et al., 2021). Such events eventually decreased the lipogenic gene expression and fatty acid synthase, essential for regulating the fatty acid synthesis and suppressing malonyl-CoA conversion to palmitate. The events reduced lipogenesis during fat storage. These findings suggest that PQQ inhibits lipogenesis.

## 4 PQQ and epigenetic obesity

Maternal obesity during pregnancy is a risk factor for programming obesity in the offspring (Reinehr and Wabitsch, 2011). Programming refers to how early life experiences, including prenatal and early postnatal nutrition, can have long-lasting effects on metabolism, body composition, and health outcomes later in life (Oben et al., 2010; Schack-Nielsen et al., 2010). The impact of maternal PQQ supplementation on offspring was investigated in mice. Pre- and postnatal supplementation of 3.8 µM of PQQ in water given to diet-induced obese mice reduced the body and hepatic fat of the offspring compared to that of the untreated obese offspring (Jonscher et al., 2017; Mandala et al., 2022). Maternal PQQ treatment also elevated beta-oxidation genes in the offspring, indicating improved lipid metabolism that decreased uterine TG accumulation (Mandala et al., 2022). PQQ treatment in mice substantially reduced steatosis induced by a Western diet (42% kcal from fat, 15% protein, 43% carbohydrates, 34%). These results demonstrate that PQQ also works epigenetically to attenuate fat accumulation and combat obesity in offspring. However, due to limited clinical studies, we stress here that PQQ is not yet intended for pregnant and lactating women, or for children, as a food supplement (Turck et al., 2017).

## 5 PQQ and the gut microbiota

The gut microbiota composition can influence obesity risk and overall health (Liu et al., 2021; Régnier et al., 2021). In mammals, obese and lean individuals have different microbiota compositions, functional genes, and metabolic activities, indicating that the gut microbiota may play a role in these phenotypes (Gérard, 2016). PQQ may benefit gut microbiota by increasing the abundance of certain beneficial bacteria. Supplementation with PQQ improved the gut microbiota composition and reduced inflammation markers associated with obesity in mice (Friedman et al., 2018). PQQ supplementation significantly reversed the diet-induced decrease in *Bacteroidetes* and increase in *Firmicutes* observed in the offspring (Figure 1B). Although few studies have specifically examined the impact of PQQ on microbiota composition or function, some gut bacteria may require PQQ for optimal function. However, more research is needed to fully understand the effects of PQQ on gut microbiota and obesity in humans.

## 6 PQQ mitigates mitochondrial dysfunction and inflammation

Obesity causes various changes in the body and contributes to health problems. Mitochondrial dysfunction and inflammation, implicated in obesity progression, are interconnected processes (de Mello et al., 2018). Existing inflammatory processes generate high levels of reactive oxygen species (ROS), resulting in oxidative stress that causes mitochondrial dysfunction, subsequently leading to decreased energy production and ROS accumulation, exacerbating oxidative damage. Moreover, mitochondrial dysfunction activates inflammatory pathways, producing pro-inflammatory cytokines and chemokines (de Mello et al., 2018). Prolonged inflammation decreases immune defense, a precursor of several diseases (Castro et al., 2017). During obesity, macrophages infiltrate and produce cytokines such as tumor necrosis factor-alpha (TNF-α), interleukin 1 beta (IL-1β), IL-18, IL-6, and interferon-gamma (IFN-γ). Secretion of these molecules, in addition to adipocyte destruction, triggers insulin resistance and metabolic syndrome development (Castro et al., 2017; Saltiel and Olefsky, 2017). Moreover, obesity activates inflammasomes such as NOD-like receptor family pyrin domain-containing 3 (NLRP3), which is involved in diseases such as non-alcoholic fatty liver disease (NAFLD) (Yu et al., 2022), Type 2 diabetes (Gora et al., 2021), and chronic kidney disease (CKD) (Ke et al., 2018)). Hence, maintaining healthy mitochondrial function and reducing inflammation are crucial strategies for promoting overall health and managing various health conditions.

In addition to improving mitochondrial biogenesis, PQQ exhibits anti-inflammatory properties. PQQ acts as an antioxidant by scavenging ROS and inhibiting lipid peroxidation (Miyauchi et al., 1999). PQQ also reduces inflammation by modulating inflammation regulators through NF-kB and MAPK pathways (Kumar and Kar, 2015). By inhibiting NF-κB activation, PQQ decreases IL-6 and TNF-α production (Liu et al., 2016). Consequently, researchers have predicted that PQQ can mitigate obesity-associated inflammation. Indeed, the serum levels of IL-18, IL-1, TNF-α, and IL-6 decreased after 5 weeks PQQ treatment in diet-induced obese rats (Devasani and Majumdar, 2019). Conversely, PQQ decreased NLRP3 inflammasome and caspase 1 expression, suggesting that PQQ managed mitochondrial dysfunction and low-grade inflammation caused by obesity by improving mitochondrial biogenesis (Devasani et al., 2020). In a mouse model study, PQQ supplementation reduced pro-inflammatory cytokine expression, particularly NLRP3, NOS-2, and IL-6. PQQ was more effective on IL-6 than on TNF and IL-1β (Jonscher et al., 2017). These results suggest that the action of PQQ on mitochondrial biogenesis is beneficial for treating mitochondrial dysfunction and inflammation caused by obesity.

## 7 PQQ and obesity-associated metabolic diseases

AMPK is a key cellular energy regulator that plays a crucial role in maintaining cellular energy homeostasis (Hardie, 2014). Given its impact on AMPK and mitochondrial function described in Section 3, PQQ has considerable potential for treating numerous metabolic diseases including diabetes, NAFLD, and CKD. Moreover, PQQ influences insulin signaling via multiple pathways and increases glucose absorption via glucose transporter translocation. Thus, studies suggest that PQQ may be beneficial in insulin resistance and type 2 diabetes (Akagawa et al., 2016). In a cell culture study, the HK-1 human proximal tubular epithelial cell line modeled the progression of diabetes in the kidney following exposure to high glucose (HG) levels. Diabetes progressing HK-1 cells had an increase in ROS and pro-inflammatory gene expression, and factor erythroid 2-related factor 2 (NFE2L2) and its target inhibition. PQQ supplementation of HG-treated HK-1 cells activated NFE2L2 signaling by increasing NFE2L2 translocation to the nucleus (Wang et al., 2019). PQQ also reduces pro-inflammatory signaling *in vivo* and *in vitro*, regulated by the NLRP3 inflammasome (Lin et al., 2020).

PQQ showed protective effects on lipotoxicity and inflammation caused by maternal obesity, and intervention on the risk of NAFLD (Jonscher et al., 2017) and CKD (Zhou et al., 2021). Maternal obesity induced the NLRP3 inflammasome activation, leading to pro-inflammatory factor secretion in the offspring. Maternal supplementation of PQQ alleviates lipotoxicity in the fetal kidney and liver by decreasing ROS levels and inhibiting inflammasome NLRP3 activation. Taken together, PQQ could mitigate the progressive development of obesity-related complications, including diabetes, NAFLD, and CKD. However, further research is needed to fully understand its mechanism of action and potential therapeutic applications in obesity.

## 8 PQQ with other drugs

Statins are antihyperlipidemic drugs prescribed to those at high risk of cardiovascular disease. Unfortunately, statin therapy is clinically limited for various reasons, including inadequate treatment outcomes (Barry et al., 2018). Furthermore, 20% of people cannot take statins due to intolerance, and 40%–75% of patients discontinue statin treatment within 1–2 years of starting treatment (Toth et al., 2018). Moreover, high statin doses worsen glycemic control in people with diabetes mellitus and increase the chances of developing the disease (Angelidi et al., 2018). The application of PQQ with atorvastatin (ATS) (10 or 20 mg/kg of both molecules) to improve clinical outcomes in obesity and associated low-grade inflammation was investigated. PQQ was expected to counteract the adverse effects of ATS, lessen its deleterious effects, and enhance metabolic results. This study was the first to examine how PQQ affects the ATS treatment results and revealed that combining PQQ with ATS reduced hyperlipidemia and inflammatory reactions and boosted mitochondrial biogenesis (Devasani et al., 2020). Although the positive action of PQQ in alleviating the adverse effects of statins was shown, more research is needed before it can be recommended as a treatment.

## 9 Related clinical studies

Although animal studies have highlighted PQQ’s potential for treating obesity, no clinical studies have been conducted. However, PQQ has been demonstrated to enhance energy metabolism in humans. When a single dose (0.2 mg/kg) daily for 3 days (0.3 mg/kg) of PQQ was administered to 10 young participants (five males and five females), the levels of trimethylamine N-oxide, a marker of perturbed energy metabolism, decreased following PQQ intake. The ratio of blood lactate to pyruvate and the profile of urinary metabolites were consistent with enhanced mitochondrial oxidation. Additionally, PQQ reduced inflammation indices, C-reactive protein, IL-6 levels, and plasma malondialdehyde levels (Harris et al., 2013). Thus, PQQ positively influences mitochondrial efficiency and attenuates human inflammation.

Another study was conducted on 29 healthy adults with normal-to-moderately high TG levels (110–300 mg/dL) to investigate the effects of PQQ on serum TG and cholesterol levels (Nakano et al., 2015). At an oral dosage of 20 mg/day for 12 weeks, LDL-cholesterol levels substantially decreased in individuals with high LDL-cholesterol (≥140 mg/dL) at baseline compared to the placebo group. However, the serum TG levels did not change. Although the sample size was small, this result regarding the benefits of PQQ for cholesterolemia is promising and warrants further investigation. Investigating the impact of PQQ on obese participants would address some of the limitations.

## 10 Conclusion

PQQ has emerged as a novel factor that contributes to obesity management. Dietary supplementation with PQQ reduced visceral and hepatic fat accumulation by enhancing mitochondria-related oxidative metabolism and suppressing lipogenesis. Notably, administering PQQ has shown beneficial effects in attenuating clinically relevant dysfunctions, such as mitochondrial dysfunction, inflammation, hyperlipidemia, and lipotoxicity. Evidence from multiple animal lines and cell cultures supports PQQ as a promising therapeutic agent for obesity and its associated complications, such as NAFLD, diabetes, and CKD. However, further research into the anti-obesity potential of PQQ is required. Overall, PQQ may facilitate obesity treatment as research in the field of study progresses.

## Abbreviations

AMPK, AMP-activated protein kinase; ATS, Atorvastatin; BBP, Benzyl butyl phthalate; CKD, Chronic kidney disease; EDC, Endocrine disrupting chemical; F/B, Firmicutes/Bacteroidetes; HFD, High-fat diet; IL, Interleukin; NAD, Nicotinamide adenine dinucleotide; NADH, Reduced form of nicotinamide adenine dinucleotide; NAFLD, Non-alcohol fatty liver disease; NFE2L2, Factor erythroid 2-related factor 2; NLRP3, NOD-like receptor family pyrin domain containing 3; PGC-1α, Peroxisome proliferator-activated receptor-gamma coactivator 1 alpha; PQQ, Pyrroloquinoline quinone; ROS, Reactive oxygen species; TG, Triglyceride; TNF, Tumor necrosis factor.
